# Predictors of Out-of-Hospital Cardiac Arrest in Patients Hospitalized With Acute Coronary Syndrome: A Systematic Review and Meta-Analysis

**DOI:** 10.7759/cureus.48609

**Published:** 2023-11-10

**Authors:** Ibrahim Reyaz, Calvin R Wei, Anurag Rawat, Eemaz Nathaniel, Morshed Alam, Abdullah Tarboush, Omair Bseiso, Neelum Ali

**Affiliations:** 1 Internal Medicine, Christian Medical College and Hospital, Ludhiana, Ludhiana, IND; 2 Research and Development, Shing Huei Group, Taipei, TWN; 3 Interventional Cardiology, Himalayan Institute of Medical Sciences, Dehradun, IND; 4 Research, Rehman Medical Institute, Peshawar, PAK; 5 Internal Medicine, Chittagong Medical College, Chittagong, BGD; 6 General Practice, Mansoura University, Mansoura, EGY; 7 Medicine, College of Medicine, Hebron University, Hebron, PSE; 8 Internal Medicine, University of Health Sciences, Lahore, PAK

**Keywords:** observational studies, systematic review and meta-analysis, myocardial infarction, out of hospital cardiac arrest, predictors

## Abstract

Out-of-hospital cardiac arrest (OHCA) refers to the abrupt stoppage of the heart's mechanical activity, primarily triggered by coronary artery disease. OHCA represents a significant global cause of death. The aim of this study was to assess the predictors of OHCA in patients admitted with acute coronary syndrome (ACS). This meta-analysis was conducted following the guidelines of the Preferred Reporting Items for Systematic Reviews and Meta-Analyses. Two investigators performed a comprehensive search of online databases, including PubMed, EMBASE, and Web of Science, from their inception to October 15, 2023. Keywords such as "predictors," "out-of-hospital cardiac arrest," and "acute coronary syndrome" were used to identify relevant articles. To enhance the search, synonyms and their corresponding Medical Subject Heading terms were included. A total of six studies were included in this meta-analysis. The pooled incidence of out-of-hospital cardiac arrest was 4% (95% confidence interval, 3%-5%). The current meta-analysis reports that age, gender, having multivessel disease, hypertension, dyslipidemia, and having ST-elevation myocardial infarction were some of the significant factors associated with OHCA in patients hospitalized with ACS.

## Introduction and background

Out-of-hospital cardiac arrest (OHCA) refers to the abrupt stoppage of the heart's mechanical activity, primarily triggered by coronary artery disease (CAD) [1]. OHCA represents a significant global cause of death [2]. In the United States, over 400,000 individuals experience OHCA each year, with approximately only 10% surviving until hospital discharge when treated by emergency medical services personnel [3]. Acute myocardial infarction (MI) often triggers OHCA, with nearly half of resuscitated OHCA patients showing a blocked coronary vessel in coronary angiography, even in some cases without initial ST-segment elevation on their electrocardiogram [4,5].

Over the past two decades, research has identified various clinical factors linked to cardiac arrest or ventricular arrhythmias in acute MI patients, including a history of hypertension and previous heart attacks, smoking, larger infarct size, low potassium levels, and significant ST-segment deviations [6-8]. However, in recent years, there has been limited epidemiological investigation into pre-hospital cardiac arrest among those with acute coronary syndrome (ACS). Moreover, recent studies have primarily focused on cardiac arrest cases occurring after hospital admission [9]. Since these initial studies, the management of post-arrest patients has improved significantly through therapeutic hypothermia, beta-blockers, angiotensin-converting enzyme (ACE) inhibitors, implantable cardiac defibrillators, and other therapies [10]. Nevertheless, there is a lack of comprehensive understanding regarding the distinct management approaches for ACS patients with and without pre-hospital cardiac arrest.

While previous studies have dedicated substantial efforts to explore the intricate characteristics and potential predictors of OHCA in conjunction with ACS within the broader landscape of cardiovascular emergencies, it is important to acknowledge that these investigations have been hindered by the relatively sparse representation of OHCA cases with ACS within the confines of their limited study populations. Consequently, the body of knowledge pertaining to the specific interplay of OHCA and ACS remains relatively uncharted and underdeveloped. Therefore, we have used previous studies to conduct pooled analysis to determine predictors of out-of-hospital cardiac arrest in patients admitted with ACS, which was the aim of this study.

## Review

Methodology

This meta-analysis was conducted following the guidelines of the Preferred Reporting Items for Systematic Reviews and Meta-Analyses (PRISMA).

Search Strategy and Study Selection

Two investigators performed a comprehensive search of online databases, including PubMed, EMBASE, and Web of Science, from their inception to October 15, 2023. Keywords such as "predictors," "out-of-hospital cardiac arrest," and "acute coronary syndrome" were used to identify relevant articles. To enhance the search, synonyms and their corresponding Medical Subject Heading (MeSH) terms were included. Furthermore, the reference lists of all included studies were manually screened to identify additional studies that were relevant to the study objective.

All records obtained from the online database searches were imported into EndNote X9 (Clarivate Analytics, Philadelphia, PA). After removing duplicates, two authors evaluated the remaining records using predefined inclusion and exclusion criteria. Full texts of eligible records were acquired and subjected to a detailed assessment. Any disagreements between the two authors during the search strategy and study selection process were resolved through discussion.

We included studies that assessed factors associated with out-of-hospital cardiac arrest in patients who were hospitalized with ACS. We included all studies regardless of the sample size and geographical location. Case reports, case series, reviews, and editorials were excluded. Additionally, studies published in languages other than English were also excluded.

Data Extraction and Quality Assessment

Data were extracted using a pre-designed data extraction sheet created in Microsoft Excel (Microsoft Corporation, Redmond, WA). One author extracted the data, and a second author cross-checked it and entered it into Review Manager (RevMan; Cochrane Collaboration, London) for data analysis. The extracted data from the included studies included author names, year of publication, region, total population, the number of patients with OHCA, and information about predictors.

The quality assessment of the studies included in our research was conducted using the Newcastle-Ottawa Scale (NOS). The NOS is a well-recognized tool for evaluating the methodological quality and risk of bias in non-randomized studies, especially in systematic reviews and meta-analyses. This scale assesses the quality of the included studies based on three key aspects: the selection of study groups, the comparability of these groups, and the ascertainment of the exposure or outcome of interest.

Data Analysis

Data analysis was carried out using RevMan, version 5.4.1 and Stata, version 18.0 (StataCorp LLC, College Station, TX). To assess the impact of continuous variables on out-of-hospital cardiac arrest, the mean difference (MD) was calculated with a 95% confidence interval (CI), while for categorical variables, odds ratios (ORs) were calculated with a 95% CI. The heterogeneity among the study results was reported as I-square (I^2^) values. I^2^ values exceeding 50% were considered as significant heterogeneity. Random-effect models were employed to account for variation among the studies.

Results

**Figure 1:**
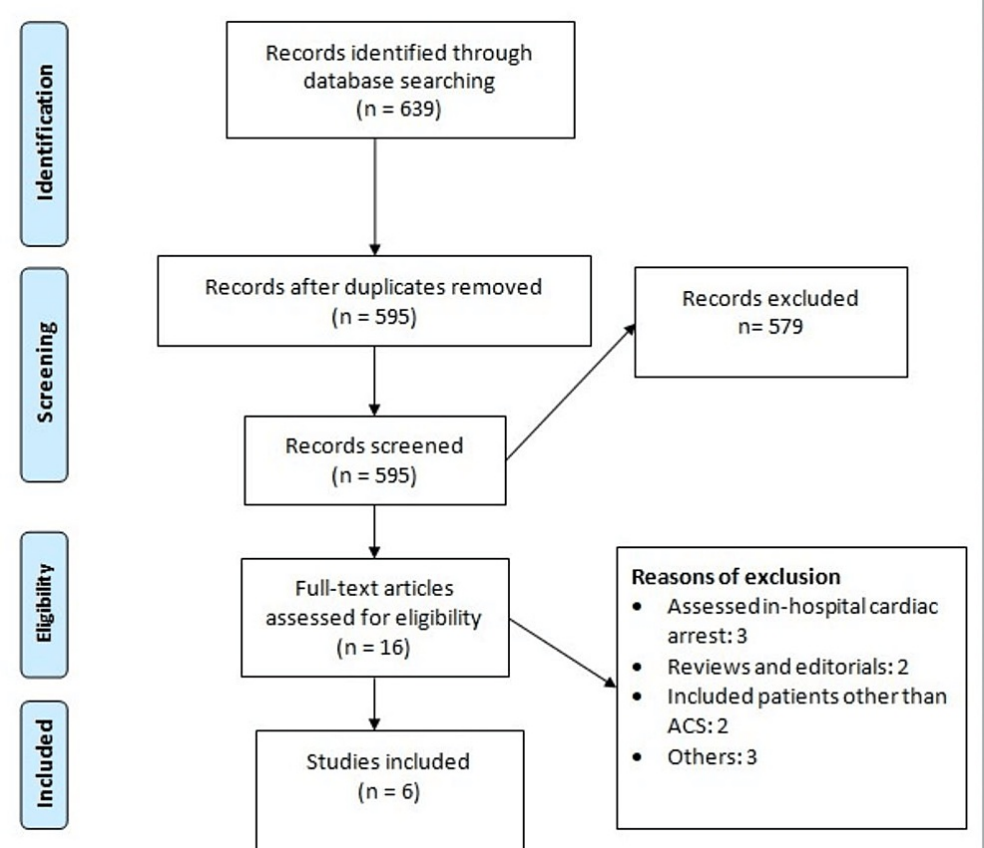
PRISMA flowchart of study selection PCS, Preferred Reporting Items for Systematic Reviews and Meta-Analyses; ACS, acute coronary syndrome

Initial electronic database searching revealed 639 records. After removing duplicates, 595 studies were initially screened using their abstracts and titles. The full text of 16 studies was obtained and detailed assessment was done based on pre-defined inclusion and exclusion criteria. Finally, six studies were included in this meta-analysis. Figure 1 shows the PRISMA flowchart of study selection. Table 1 shows the characteristics of included studies. The pooled incidence of OHCA was 4% (95% CI, 3%-5%). Table 2 shows the quality assessment of included studies.

**Table 1:** Characteristics of included studies OHCA, out-of-hospital cardiac arrest

Author name	Year	Region	Sample size	No. of OHCA cases
Alsaeed et al. [11]	2023	Saudi Arabia	10,848	188
Dawson et al. [12]	2020	Australia	12,637	1057
Faxén et al. [13]	2020	Sweden	121,379	349
Fordyce et al. [14]	2016	Canada	54,860	641
Kosugi et al. [15]	2020	Japan	480	141
Li et al. [16]	2013	Canada	14,010	206

**Table 2:** Quality assessment of included studies

Author name	Selection	Group comparability	Outcome	Overall
Alsaeed et al. [11]	2	2	3	Good
Dawson et al. [12]	3	2	3	Good
Faxén et al. [13]	3	1	2	Fair
Fordyce et al. [14]	2	2	3	Good
Kosugi et al. [15]	3	1	2	Fair
Li et al. [16]	2	2	3	Good

Predictors of OHCA in Patients Hospitalized With ACS

Table 3 presents the results of a pooled analysis of factors associated with OHCA in patients hospitalized with ACS. The mean age of patients with OHCA was significantly lower compared to those without OHCA (MD, -2.62; 95% CI, -3.92 to -1.32). Additionally, gender showed a significant association with OHCA. As indicated in Table 3, the odds of being male were significantly higher in OHCA patients compared to non-OHCA patients (OR, 1.71; 95% CI, 1.53-1.92).

**Table 3:** Predictors of out-of-hospital cardiac arrest OR, odds ratio; CI, confidence interval; BMI, body mass index; CABG, coronary artery bypass graft; ACS, acute coronary syndrome; STEMI, ST-elevation myocardial infarction OR>1 shows the number is greater in OHCA patients compared to non-OHCA patients, and OR<1 shows the number is greater in non-OHCA patients. *Mean difference (95% CI) ^Significant at p<0.05

Variable	OR (95% CI)	I^2^
Age*	-2.62 (-3.92 to -1.32)^	89%
Gender (male)	1.71 (1.53 to 1.92)*	13%
BMI*	-0.11 (-0.34 to 0.12)	0%
Diabetes	0.92 (0.69 to 1.23)	88%
Hypertension	0.58 (0.39 to 0.85)^	94%
Hyperlipidemia	0.60 (0.47 to 0.76)^	83%
Heart failure	1.18 (0.49 to 2.88)	95%
CABG	1.36 (0.34 to 5.45)	99%
Type of ACS (STEMI)	2.78 (1.52 to 5.06)^	96%
Multivessel disease	1.41 (1.03 to 1.91)^	80%

In terms of body mass index (BMI), no significant difference was observed between the two groups (MD, -0.11; 95% CI, -0.34 to 0.12). The odds of individuals having diabetes did not significantly differ between patients with OHCA and those without OHCA (OR, 0.92; 95% CI, 0.69-1.23). However, the odds of hypertension were significantly higher in patients without OHCA compared to those with OHCA (OR, 0.58; 95% CI, 0.39-0.85). Similarly, the odds of dyslipidemia were significantly higher in patients without OHCA compared to those with OHCA (OR, 0.60; 95% CI, 0.47-0.76).

As shown in Table 3, no significant difference was observed between OHCA and non-OHCA patients in terms of heart failure and a past history of coronary artery bypass graft (CABG). Furthermore, the odds of ST-elevation myocardial infarction (STEMI) were significantly higher in patients with OHCA compared to non-OHCA patients (OR, 2.78; 95% CI, 1.52-5.06). Similarly, the odds of multi-vessel disease were significantly higher in patients with OHCA compared to those without OHCA (OR, 1.41; 95% CI, 1.03-1.91).

We compared four medications including beta blockers, aspirin, calcium channel blockers and statin between OHCA and non-OHCA patients. As shown in Table 4, the number of patients taking aspirin, statins and calcium channel blockers was significantly higher in the non-OHCA group compared to patients who developed OHCA. However, the number of patients taking beta-blockers was not significantly different between two groups.

**Table 4:** Comparison of drugs between OHCA and non-OHCA patients OHCA, out-of-hospital cardiac arrest; OR, odds ratio; CI, confidence interval OR>1 shows the number is greater in OHCA patients compared to non-OHCA patients, and OR<1 shows the number is greater in non-OHCA patients. ^Significant at p<0.05

Medication	OR (95% CI)	I^2^
Aspirin	0.51 (0.36 to 0.71)^	68%
Statins	0.57 (0.33 to 0.99)^	91%
Beta-blockers	0.84 (0.46 to 1.55)	91%
Calcium-channel blockers	0.53 (0.37 to 0.76)^	61%

Discussion

As far as our knowledge is concerned, this is the first meta-analysis to determine the incidence and predictors of OHCA in patients hospitalized with ACS. The current meta-analysis explored multiple factors, including age, gender, BMI, diabetes, hypertension, dyslipidemia, heart failure, history of CABG, and the type of ACS. The current meta-analysis reports that age, gender, having multivessel disease, hypertension, dyslipidemia, and having STEMI were some of the significant factors associated with OHCA in patients hospitalized with ACS.

Cardiac arrest is one of the most common causes of death in the early phase of acute ACS. The prevalence of cardiac arrest at any time after discharge from the hospital in the included studies ranged from 0.28% to 29.37%. The pooled incidence of cardiac arrest reported in the present meta-analysis was 4% (95% CI, 3%-5%).

In this study, we found that older patients with ACS had a lower risk of OHCA compared to young individuals. Among all the six included studies, five reported that the mean age of patients who experienced OHCA was significantly lower compared to those without OHCA. Previous studies have also reported that older patients with myocardial infarction had unexpectedly better prognoses once they were discharged from healthcare facilities to skilled nursing facilities or home, even though they had a higher prevalence of heart failure, major bleeding, and in-hospital cardiogenic shock [17-18].

The current meta-analysis reports that the likelihood of cardiac arrest is greater in males compared to females. All included studies supported this claim. One of the possible reasons for the high incidence of cardiac arrest in males in all included studies is the underrepresentation of females, as in all studies, the number of males was higher compared to females. While there is data indicating that estrogen may play a protective role in the heart, brain, and kidneys, our understanding of its function in these organs remains incomplete. Female patients experience higher mortality from OHCA, highlighting the need for sex-specific research [19].

In addition, the number of STEMI patients was significantly higher in patients with OHCA compared to their counterparts. It is particularly concerning that OHCA rates are often the highest among patients diagnosed with STEMI. This phenomenon can be attributed to several interrelated factors. First, STEMI is a type of heart attack caused by a complete blockage of a coronary artery, leading to a significant and abrupt decrease in blood flow to the heart muscle [20]. This lack of blood supply can quickly result in lethal arrhythmias, which can ultimately lead to cardiac arrest. Additionally, the underlying coronary artery disease in STEMI patients, often involving multiple vessels, puts them at a heightened risk of cardiac events [21]. Furthermore, the nature of STEMI itself makes it a more acute and life-threatening condition, increasing the chances of sudden cardiac arrest. Therefore, the combination of severe coronary artery disease, the rapid onset of heart damage, and the potential for lethal arrhythmias makes STEMI patients particularly vulnerable to OHCA, necessitating prompt and effective emergency care to improve their chances of survival [22].

Notably, in the current meta-analysis, the utilization of calcium-channel blockers was significantly linked to the absence of OHCA occurrence. Only three of the included studies assessed the association between calcium-channel blockers and OHCA within the context of acute coronary syndrome, and all three studies reported a significantly higher usage of these drugs in non-OHCA patients. The absence of calcium-channel antagonist usage could indicate a lack of medical treatment in general; nevertheless, it is also plausible that calcium-channel antagonists might prevent OHCA by hindering vasospasm or life-threatening arrhythmias. It is well-established that coronary spasms frequently occur in locations with substantial atherosclerosis and significant plaque buildup [23-24]. Statins have anti-inflammatory effects, and inflammation is closely linked to the progression of atherosclerosis and plaque rupture. By reducing inflammation within coronary arteries, statins may prevent acute coronary events and OHCA [25]. Aspirin's antiplatelet effects can reduce the risk of acute coronary events, which can lead to OHCA. By keeping blood vessels more open and preventing clot formation, aspirin may lower the risk of severe cardiac events that can culminate in OHCA [26]. The present study also reported a lower likelihood of OHCA in patients receiving statins and aspirin. However, future studies are needed to validate these findings and understand how these drugs can affect outcomes post-ACS to develop guidelines and recommendations for healthcare professionals.

The current meta-analysis has certain limitations. First, only two studies assessed angiographic findings and their impact on the incidence of OHCA, and out of these two, only one performed detailed angiographic assessment. In the future, more studies are required to assess how different angiography findings, such as proximal lesions and bifurcations, affect OHCA in ACS patients. Second, only six studies were included in this meta-analysis. Future studies are needed to better understand the epidemiology of OHCA in patients hospitalized with ACS.

## Conclusions

It was found that the pooled incidence of out-of-hospital cardiac arrest in patients hospitalized with acute coronary syndrome was 4%. Various predictors of OHCA were identified, including age, gender, the presence of multivessel disease, hypertension, dyslipidemia, and the diagnosis of ST-elevation myocardial infarction. The absence of calcium-channel blocker usage was linked to OHCA, suggesting potential preventative effects. Statins and aspirin also showed promise in reducing the OHCA risk. However, further research is needed to validate these findings and establish treatment guidelines.

## References

[1] Rubart M, Zipes DP (2005). Mechanisms of sudden cardiac death. J Clin Invest.

[2] Sasson C, Rogers MA, Dahl J, Kellermann AL (2010). Predictors of survival from out-of-hospital cardiac arrest: a systematic review and meta-analysis. Circ Cardiovasc Qual Outcomes.

[3] Go AS, Mozaffarian D, Roger VL (2014). Heart disease and stroke statistics—2014 update: a report from the American Heart Association. Circulation.

[4] Spaulding CM, Joly LM, Rosenberg A, Monchi M, Weber SN, Dhainaut JF, Carli P (1997). Immediate coronary angiography in survivors of out-of-hospital cardiac arrest. N Engl J Med.

[5] Kern KB, Lotun K, Patel N (2015). Outcomes of comatose cardiac arrest survivors with and without ST-segment elevation myocardial infarction: importance of coronary angiography. JACC Cardiovasc Interv.

[6] Flugelman MY, Hasin Y, Tur-Caspa I, Friedlander Y, Gotsman MS (1983). Prediction of in-hospital ventricular fibrillation from admission data in acute myocardial infarction. Clin Cardiol.

[7] Nordrehaug JE, Johannessen KA, von der Lippe G (1985). Serum potassium concentration as a risk factor of ventricular arrhythmias early in acute myocardial infarction. Circulation.

[8] Fiol M, Marrugat J, Bayes A, Bergadá J, Guindo J (1993). Ventricular fibrillation markers on admission to the hospital for acute myocardial infarction. Am J Cardiol.

[9] McManus DD, Aslam F, Goyal P, Goldberg RJ, Huang W, Gore JM (2012). Incidence, prognosis, and factors associated with cardiac arrest in patients hospitalized with acute coronary syndromes (the Global Registry of Acute Coronary Events Registry). Coron Artery Dis.

[10] Pell JP, Corstorphine M, McConnachie A (2006). Post-discharge survival following pre-hospital cardiopulmonary arrest due to cardiac aetiology: temporal trends and impact of changes in clinical management. Eur Heart J.

[11] Alsaeed AH, Hersi A, Kashour T (2023). Characteristics and predictors of out-of-hospital cardiac arrest in young adults hospitalized with acute coronary syndrome: a retrospective cohort study of 30,000 patients in the Gulf region. PLoS One.

[12] Dawson LP, Dinh D, Duffy S (2020). Short- and long-term outcomes of out-of-hospital cardiac arrest following ST-elevation myocardial infarction managed with percutaneous coronary intervention. Resuscitation.

[13] Faxén J, Jernberg T, Hollenberg J, Gadler F, Herlitz J, Szummer K (2020). Incidence and predictors of out-of-hospital cardiac arrest within 90 days after myocardial infarction. J Am Coll Cardiol.

[14] Fordyce CB, Wang TY, Chen AY (2016). Long-term post-discharge risks in older survivors of myocardial infarction with and without out-of-hospital cardiac arrest. J Am Coll Cardiol.

[15] Kosugi S, Shinouchi K, Ueda Y (2020). Clinical and angiographic features of patients with out-of-hospital cardiac arrest and acute myocardial infarction. J Am Coll Cardiol.

[16] Li Q, Goodman SG, Yan RT (2013). Pre-hospital cardiac arrest in acute coronary syndromes: insights from the Global Registry of Acute Coronary Events and the Canadian Registry of Acute Coronary Events. Cardiology.

[17] Lettieri C, Savonitto S, De Servi S (2009). Emergency percutaneous coronary intervention in patients with ST-elevation myocardial infarction complicated by out-of-hospital cardiac arrest: early and medium-term outcome. Am Heart J.

[18] Awad HH, Anderson FA Jr, Gore JM, Goodman SG, Goldberg RJ (2012). Cardiogenic shock complicating acute coronary syndromes: insights from the Global Registry of Acute Coronary Events. Am Heart J.

[19] Jarman AF, Mumma BE, Perman SM, Kotini-Shah P, McGregor AJ (2019). When the female heart stops: sex and gender differences in out-of-hospital cardiac arrest epidemiology and resuscitation. Clin Ther.

[20] Kosmopoulos M, Bartos JA, Yannopoulos D (2021). ST-elevation myocardial infarction complicated by out-of-hospital cardiac arrest. Interv Cardiol Clin.

[21] Canto JG, Kiefe CI, Rogers WJ (2011). Number of coronary heart disease risk factors and mortality in patients with first myocardial infarction. JAMA.

[22] Amsterdam EA, Wenger NK, Brindis RG (2014). 2014 AHA/ACC Guideline for the Management of Patients With Non-ST-Elevation Acute Coronary Syndromes: a report of the American College of Cardiology/American Heart Association Task Force on Practice Guidelines. J Am Coll Cardiol.

[23] Sunagawa O, Shinzato Y, Touma T, Tomori M, Fukiyama K (2000). Differences between coronary hyperresponsiveness to ergonovine and vasospastic angina. Jpn Heart J.

[24] Zeiher AM, Schächlinger V, Hohnloser SH, Saurbier B, Just H (1994). Coronary atherosclerotic wall thickening and vascular reactivity in humans. Elevated high-density lipoprotein levels ameliorate abnormal vasoconstriction in early atherosclerosis. Circulation.

[25] Yu M, Tsai SF, Kuo YM (2017). The therapeutic potential of anti-inflammatory exerkines in the treatment of atherosclerosis. Int J Mol Sci.

[26] Dai Y, Ge J (2012). Clinical use of aspirin in treatment and prevention of cardiovascular disease. Thrombosis.

